# Supplementary material for: ITRAQ Based Proteomics Reveals the Potential Mechanism of Placental Injury Induced by Prenatal Stress
Source: Int J Mol Sci. 2024 Sep 16;25(18):9978. doi: 10.3390/ijms25189978 (PMC11432224; doi:10.3390/ijms25189978)
Supplement: Supplementary file 1 [file ijms-25-09978-s001.zip › ijms-3139614-supplementary.pdf]

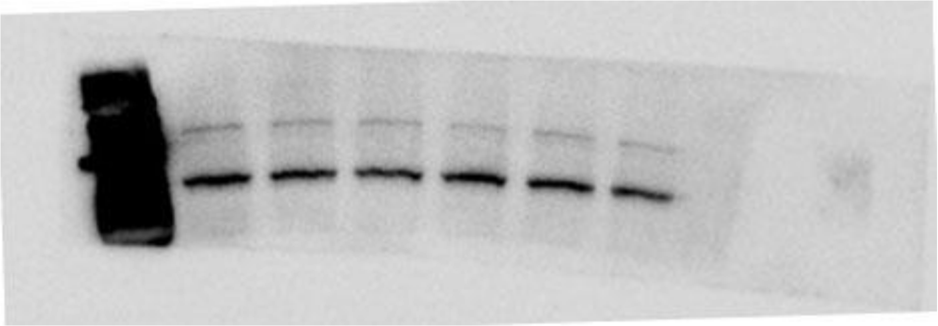

1. Representative western blotting image of PI3K was shown

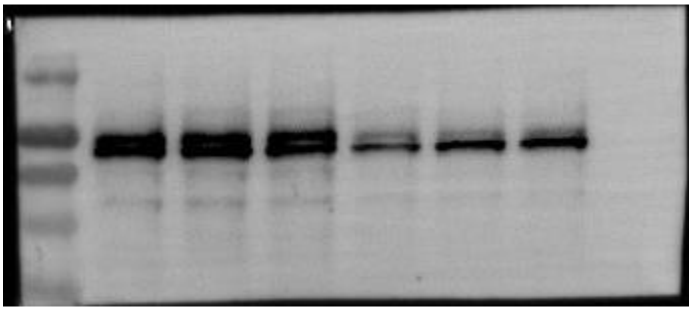

2. Representative western blotting image of AKT was shown

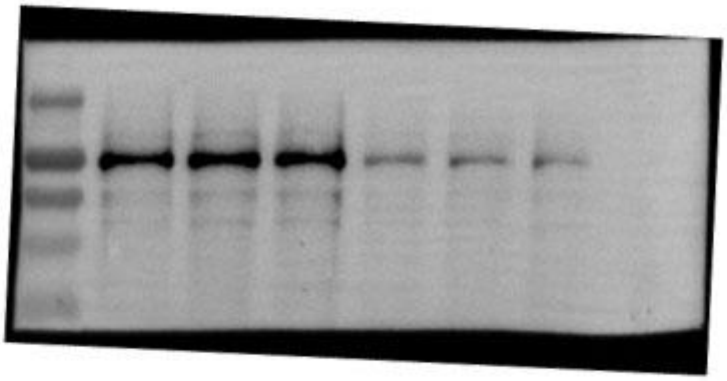

3. Representative western blotting image of p-AKT was shown

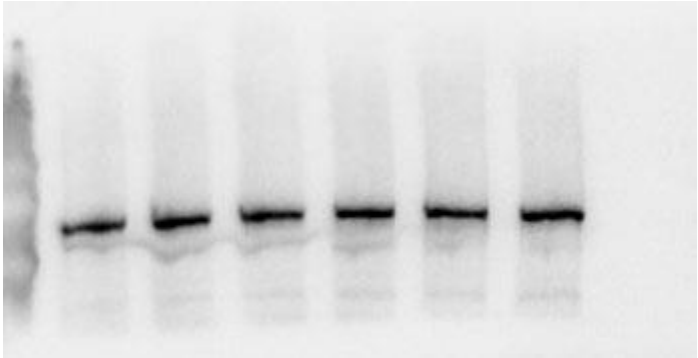

4. Representative western blotting image of mTOR was shown

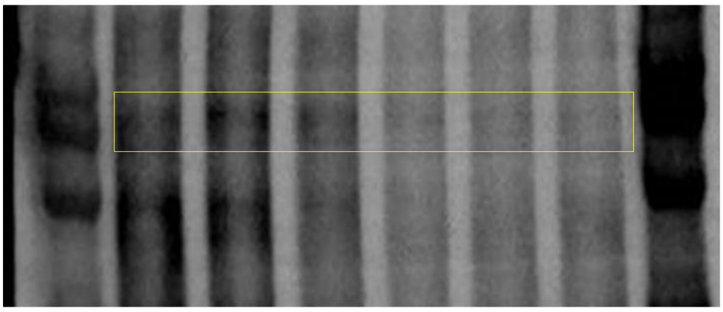

5. Representative western blotting image of p-mTOR was shown

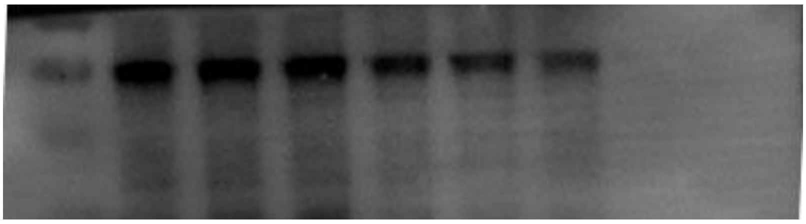

6. Representative western blotting image of CDC37 was shown

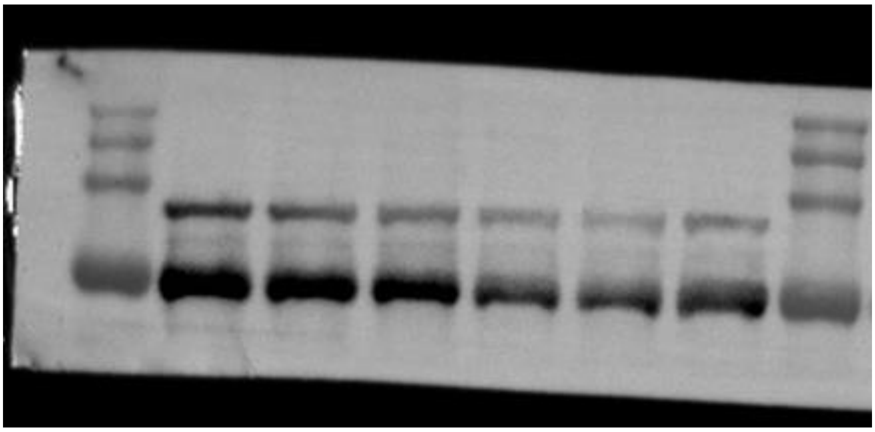

7. Representative western blotting image of HSP90bata was shown

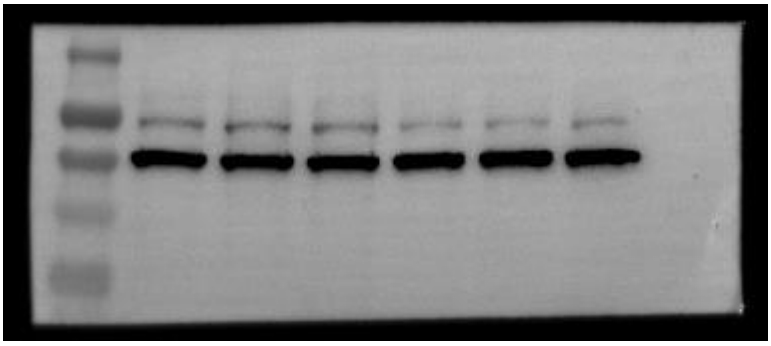

8. Representative western blotting image of α -tubulin was shown
